# Serum electrolytes disorder and its associated factors among adults admitted with metabolic syndrome in Jimma Medical Center, South West Ethiopia: Facility based crossectional study

**DOI:** 10.1371/journal.pone.0241486

**Published:** 2020-11-05

**Authors:** Abebe Timerga, Endryas Kelta, Chala Kenenisa, Belay Zawdie, Aklilu Habte, Kassahun Haile

**Affiliations:** 1 Department of Biomedical Science, Wolkite University, Biochemistry unit, College of Medicine and Health Sciences, Wolkite, Southern Ethiopia; 2 Department of Biomedical Science, Institute of Health, Jimma University, Biochemistry unit, Faculty of Medical Sciences, Jimma, South West Ethiopia; 3 Reproductive Health Unit, School of Public Health, College of Medicine and Health Sciences, Wachamo University, Hosanna, Southern Ethiopia; 4 Department of Medical laboratory Science, College of Medicine and Health Science Wolkite University, Hematology unit, Wolkite, Southern Ethiopia; Addis Ababa University, ETHIOPIA

## Abstract

**Background:**

Electrolytes play an important role in controlling acid base balance, blood clotting, and body fluid and muscle contractions. Serum electrolytes concentrations are most commonly used tests for assessment of a patient’s clinical conditions, and are associated with morbidity and mortality. Any derangements from the normal range of electrolyte levels in the body is described as electrolyte disorders. The Current study was aimed to determine serum electrolytes disorder and its associated factors among adults admitted with metabolic syndrome at Jimma medical center, South West Ethiopia.

**Methods:**

A Facility based cross sectional study was conducted on 256 patients admitted medical center with metabolic syndrome during the study period. The World Health Organization stepwise assessment tools and patients’ medical records were used to collect information on factors associated with electrolyte disorders. Bivariable and Multivariable logistic regression analyses were performed to identify factors associated with electrolyte disorder at the level of significance of p value <0.25 with 95% confidence interval of crude odds ratio and <0.05 with 95% confidence interval of adjusted odds ratio respectively.

**Results:**

The overall prevalence of electrolyte disorders was 44.1% (95%CI:40.99–47.20) with hyponatremia 42.9% (95%CI:39.81–45.99) as the leading electrolyte disorder followed by hypokalemia 20.7% (95%CI:18.17–23.23), hypochloremia 17.6% (95%CI:15.22–19.98) and hypocalcemia 9.4% (95%CI:7.57–11.22). Non-formal education [AOR: 6.81; 95%CI:(3.48,17.01)] alcohol consumption [AOR: 4.28; 95%CI:(1.71,10.70)], diuretics, diuretics [AOR: 4.39; 95%CI:(2.10,9.15)], antidiabetics [AOR: 5.18; 95%CI:(2.44,11.00)], and body mass index [AOR: 11.51; 95%CI:(3.50,18.81)] were identified as independent factors for electrolyte disturbance in multivariable logistic regression.

**Conclusion:**

The finding of the study revealed that nearly half the study participants with metabolic syndromes had electrolyte disorder. Educational status, habit of alcohol consumption, diuretics, antidiabetics, and having higher body mass index were the independent factors associated with electrolyte disorders. Determination of Serum electrolytes, proper administration of diuretic and health education on behavioral factors were the necessary measures that should be done by concerned bodies to reduce electrolytes disorder.

## Introduction

In physiology, electrolytes are electrically charged molecules which play an important role in controlling acid base balance, blood clotting, and body fluid and muscle contractions. There are several common electrolytes found in the body, each serving a specific and important role, but most are in some part responsible for maintaining the balance of fluids between the intracellular and extracellular environments. This balance is critically important for issues like hydration, nerve impulses, and muscle function and pH level. The major electrolytes in the body are Potassium, Sodium, Magnesium, Calcium and Chloride. Serum Sodium, Potassium and Chloride are considered as the major determinants of electrophysiological properties of myocardial membrane [1,2].

Electrolyte disorders are common in clinical practice and mainly encountered in hospital populations occurring in a broad spectrum of patients (from asymptomatic to critically ill) and being associated with increased morbidity and mortality [1]. Various pathophysiological factors, such as nutritional status, gastrointestinal absorption capacity, coexistent acid-base abnormalities, pharmacological agents, other co-morbid diseases (mainly renal disease) or acute illness, alone or in combination, play a key role [3]. The relationship between metabolic syndrome (MetS) and electrolytes is complex and electrolyte imbalance may affect the course of metabolic syndromes and their management [3].

Electrolyte imbalance is common in patients with MetS which could be the result of an altered distribution of electrolytes due to hyperglycemia induced osmotic fluid shifts or of total-body deficits brought about by osmotic diuresis, in turn increases cardiovascular risks [4]. MetS encompasses a group of clinical and laboratory diagnostic test abnormalities, that are associated with a set of undesirable conditions, rooted in one’s poor lifestyle [5].

Electrolyte disorders cause substantial morbidity and mortality, and ironically, incorrect treatment can add to the problem [6]. The commonest causes of electrolyte disorders are hyperglycemia, primary Na^+^ loss due to sweating, gastrointestinal loss due to vomiting and diarrhea; renal loss due to diuretics, salt wasting nephropathy, due to a primary water gain (water retention alone) like Syndrome of Inappropriate Anidiuretic Hormone (SIADH), hypothyroidism, primary polydipsia and primary Na^+^ gain due to heart failure, hepatic cirrhosis, nephrotic syndrome and chronic renal failure [7].

Majority of the hypokalemic patients have serum potassium concentrations between 3.0 and 3.5 mmol per liter [8]. In patients with heart failure, acute aortic dissection, or diabetes mellitus, dyskalemia is associated with increased mortality risk [9] by producing life-threatening arrhythmias, flaccid paralysis, dyspnea, tetany and rhabdomyolysis [10,11].

In general, as various literature revealed, electrolyte imbalance is associated with increased mortality and morbidity as various literatures revealed. Even though, knowing the status of electrolyte disorders and associated factors in patients with metabolic syndrome is crucial, the problems were not well studied, especially in Ethiopia and scanty data with conflicting findings are available on the electrolyte profile of MetS patients. Therefore, the aim of this study was to determine magnitude of electrolyte disturbance and its associated factors among patients admitted with metabolic syndrome in Jimma Medical Center, 2019.

## Methods

### Study setting and design

A facility based cross-sectional study was conducted from April to May 2020 among patients admitted with metabolic syndromes in Jimma Medical Center which is located 352km south west of Addis Ababa city, the seat of Federal Democratic Republic of Ethiopia.

### Populations of the study

The Source populations comprised of all patients who were admitted to Jimma Medical center with MetS and the study population comprised of all selected patients with MetS who were admitted to Medical ward and who fulfilled eligibility criteria during the study period.

All admitted adult patients (≥18 years) with- Central obesity (defined as waist circumference ≥ 102cm for men and ≥ 88 cm for women) were included in the study and those admitted patient who doesn’t fulfils NCEP-ATP III major criteria (triglyceride level <150 mg/dL, HDL-C >40 mg/dL in males, >50 mg/dL in females: systolic BP <130 or diastolic BP <85 mm Hg, fasting plasma glucose <110 mg/dLand MetS patients with malignancy, pregnant mothers, patient with chronic kidney disease those patients who critically ill during data collection period were excluded from the study.

### Sample size determination and sampling techniques

The sample size was calculated by using a single population proportion formula using statcalc menu of Epi-info software version 7 by considering the following assumptions: expected frequency (the prevalence of hyponatremia among MetS patients = 29% [12], 95% confidence level, 5% degree of precision, and non-response rate of 10% which gives 316. Finally, population correction formula was employed since the population is less than 10,000 (i.e. 862) and the final sample size for the study was 232. All consecutively admitted patients who fulfilled the inclusion criteria was enrolled in the study.

### Data collection methods and procedure

A pre-tested interviewer administered structured questionnaire was developed by reviewing of relevant literatures with reasonable modifications [4,12–15]. The WHO’s stepwise (STEPs) approach for non-communicable disease surveillance are adopted and related literatures were used to collect data on NCD [16].

#### Anthropometry measurements

Body weight was measured two times at pre-stage and end of fasting by a digital scale (Seca, Hamburg, Germany) to the nearest 100g, placed in flat surface. Subjects were weighed barefoot in very light clothing. Standing height was measured with an adjustable wooden measuring board once, without shoes to the nearest 0.1 cm with the shoulders in relaxed position, arms hanging freely, feet together, heels against the back board and knees straight. Body Mass Index was calculated by dividing weight (kg) by height squared (m^2^) [17,18]. Waist circumference was measured at the midpoint between the lower margin of the least palpable rib and the top of the hip or minimal waist using stretch-resistant tape. The cut-off point for waist circumference ≥ 102cm for men and ≥ 88 cm for women were used to indicate central obesity.

#### Physical/clinical examination

Blood pressure was measured digitally (Micro life BP A50, Micro life AG, Switzerland). The BP was taken using a mercury sphygmomanometer from the right upper arm after the subject was seated quietly for 5 min [8].

#### Specimen collection and handling

After obtaining verbal consent from caregivers to participate in the study, 5mls of blood was collected by using standard serum separator tube from each participant by trained laboratory technologist through aseptic/sterile technique for the serum electrolyte determination. The drawn sample was stayed for 30 minutes and serum was separated from collected blood sample by centrifugation at 3000 rpm for 10 minutes using Rotanta 960 centrifuge in thermo stable condition. Then serum was taken and stored under -20 degree centigrade till the time of biochemical analysis. Measurement of Serum electrolytes *(Na*^*+*^, *K*^*+*^, *Ca*^*2+*^ *and Cl*^*-*^*)* was done at Jimma medical center Laboratory section by HumaLyte Plus^5^ ion selective electrolyte analyzer which was made in Germany following standard operation system (SOP). The HumaLyte ISE electrolyte analyzer system features direct ISE technology can test many ISE parameters, and maintenance is automatic washing and rinsing. It is a bench top analyzer work well in any lab, featuring a closed system with an under 60 second per sample analysis time. Na, K, Cl, iCa, and pH are included. It requires sample volume of 150 μl and auto sampler for STAT and 20 samples.

### Assay of electrolytes (Na^+^, K^+^, Ca^2+^ and Cl^-^)

Serum electrolyte was analyzed based on the ion-selective electrode method. Different electrodes were used in the analyzer: PH/Na^+^/Cl^-^, K^+^, Ca^2+^and a reference electrode. Each electrode has an ion-selective membrane that interacts specifically with the corresponding ions contained in the sample.

The procedure was started by switching on the HumaLyte Plus^5^ ion selective electrolyte analyzer, a brief countdown began. After countdown was completed, the prompt ‘Open Sample Door Introduce Sample’ will be displayed. The door will be lifted and serum sample will be put by SST, then sample door will be closed, the pump will be started to aspirate 150μl of sample by electrolyte analyzer probe (auto-sample) and the electrolyte results will be displayed. The international Federation of Clinical Chemistry (IFCC) recommended cut off values to categorize electrolyte values above and below normal range. Accordingly, the reference interval for sodium concentration level in the blood is136–145millimole per liter (mmol/L) and those study subjects whose serum sodium concentration level below 135 mmol/L and greater than 145 mmol/L were considered to be hyponatremic and hypernatremic respectively.

The reference interval for both chloride and potassium concentration levels in the blood are 98–107 mmol/L and 3.5–5.1 mmol/L respectively. MetS patients with serum chloride concentration level less than 98 mmol/L and above 107mmol/L were considered to be hypochloremic and hyperchloremic respectively. Similarly, those study subjects with calcium serum concentration level below 1.12 mmol/L and above 1.14 mmol/L were considered to be hypokalemic and hyperkalemic respectively [19].

Measurement of serum glucose total cholesterol, TG, LDL-C and HDL-C was done using fully automated, ABX Pentra 400 clinical chemistry analyzer according to the reagent manufacturer’s instruction in Jimma specialized teaching hospital (Annex IV).

### Data quality management

All investigators supervised data and specimen collectors, directly involve, and control any kind of procedures and processes that may affect the result. The specimen was collected, stored and transported according to the guideline and the suspected specimen in terms of poor quality was rejected automatically. Working and acceptable commercial kits were used. The daily performances were reported to supervisors and checked and cross checked timely. Measuring instruments and biochemical analyzers were calibrated by their respective reference materials. Two days training on the contents of the questionnaire, data collection techniques, and research ethics was given for those assistants and any doubts/question in the method they were undertook and clarified. Pretest of the questionnaire was conducted in 5% study subjects at Shenen Gibe Hospital; Jimma Zone for validation of questionnaire two weeks prior to actual data collection and some adjustment on additional preparations was done.

After checking the expiry date of both the reagents and controls, HumaLyte Plus5 ion selective electrolyte analyzer and ABX Pentra 400 clinical chemistry analyzer (Horiba ABX SAS, Montpellier, France) was checked for delivering correct result by using normal and pathological controls. Before any patient sample processed, dual quality controls (normal and pathological) was performed and the patient result was taken after the controls passed. All necessary procedures and steps were followed based on the manufacture instructions. Collected results was checked for completeness on daily basis by the principal investigator.

### Data analysis

The data were coded, cleaned, and entered by EpiDta3.1 and exported to statistical package for social science (SPSS) version 23.0 for further analysis. Descriptive statistics like frequency distributions, mean, median and standard deviation were computed. After complete entry of all the data, soft copy will be checked with its hard copy to see the consistency. After cross checking, cleaning was made to avoid missing values, outliers and inconsistencies was removed before analysis. Both bivariate and multivariable logistic regression analyses were performed to identify associations between dependent and independent variables. Crude and adjusted odds ratio with their 95% CI were calculated to determine the strength and presence of association. Variables having p value <0.25in the bivariate analysis were candidate for multivariable logistic regression. Factors significantly associated with electrolyte disturbance in the final model were identified at p value <0.05 with 95% CI of AOR. The Hosmer-Lemeshow test was used to check the appropriateness of the model for analysis. The results were presented by using tables, figures, and texts.

### Variables

The dependent variable was electrolyte disorders and the independent variables were socio demographic factors (Age, sex, educational status, employment status, monthly income and residence), behavioral characteristics (physical activity, sedentary behavior, cigarette smoking and alcohol consumption), medication related characteristics (diuretics, Antidiabetics, ACE-inhibitors, Ca-channel blockers) and anthropometric variables include; height, weight, body mass index and Waist circumference).

In this study, Electrolyte disorder means blood test results indicate an altered potassium concentration less than 3.5mEq/l or >5.1MEq/L OR chloride level <97 MEq/L or>107 MEq/L OR sodium level<135 MEq/L or>145 MEq/L OR calcium level<1.12 MEq/L or>1.14 MEq/L manifested [19]. Metabolic syndrome means entral obesity (defined as waist circumference ≥ 102cm for men and ≥ 88 cm for women); Plus, any 2 of the following 4 factors: 1) Raised triglyceride level ≥ 150 mg/dL, 2) Reduced HDL-C < 40 mg/dL in males, < 50 mg/dL in females, 3) Raised BP: systolic BP ≥ 130 or diastolic BP ≥ 85 mm Hg and 4) Raised fasting plasma glucose ≥ 110mg/dL factors [5].

Physical activity implies participants who were involved in moderate physical activities such as walking, cycling or doing that had significant benefits for health with expending energy [20]. Smoker indicates a respondent who had history of smoking of one or more manufactured or hand rolled tobacco during study period. Alcohol users showed a respondent who drinks more than 3–4 units for male and more than 2–3 units for female daily at the start of the study which results distortions in thinking [21].

In addition, chat chewing indicates a respondent who uses chat in any form during the study period [22]. Insufficient intake was to mean a respondent with inadequate intake of energy and nutrients to meet the need due to disease burden. Fruit and vegetable consumption indicated that a respondent who consumes fruit and vegetables at least once per day [23].

### Ethical approval and consent to participate

Ethical clearance was obtained from Jimma University IRB/committee; concerned administrative offices were communicated with formal letter. After getting permission, written consent was obtained from each study participant. Respondents were informed about the purpose and procedure of the study and oral consent was obtained from participants. Confidentiality of the information was assured and privacy of the respondent was maintained by keeping their information anonymous. Verbal informed consent was obtained from each study subject. Based on laboratory result, study participant at severe form of electrolyte derangements were referred to the physicians for further care and treatment.

### Results

From the total of 256 sampled patients admitted to medical ward with metabolic syndrome, all 256 were participated in the study which yielded a response rate of 100%. The patient age was ranged from 20–75 with average of 50.3 ± 13.1 years. Majorities (55.8%) of respondents were 50 years and above. With respect to level of education status, 92(35.9%) of respondents had no formal education. Almost half (49.5%) of the respondents were Orthodox by religion and Oromo, 222(86.7%) was the predominant ethnic group. Majority 211 (82.4%) of the respondents were married and nearly two fifth (38.3%) were farmers.

The average height of patients was 1.64 ± 0.07 meters with range between 1.45–1.83m and their weight ranges from 45–115 kg with mean of 71.4±9.20 kg. In addition, the average waist circumference of the patients was 98.8 ±0.5 cm with range of 88–130 cm. Waist circumference of male and female patients was 108.4cm and 94.3 cm respectively. Among a total of 256 patients, 229 (89.5%) were diabetic, 165 (64.5%) were hypertensive and 115(44.9%) were dyslipidemic **(**Table 1**).**

**Table 1:** Distribution of Socio-demographic, anthropometric and clinical characteristics among patients admitted to medical ward with metabolic syndromes, in Jimma Medical Center, South west Ethiopia from April-May 2019.

Variables	Categories	Frequency with (n = 256)	Percentage
Age	> = 50	143	55.9
	25–49	92	35.9
	<25	21	8.2
Sex	Female	123	48.1
	Male	133	51.9
Residence	Urban	123	48.1
	Rural	133	51.9
Occupational status	Farmer	98	38.3
	Daily laborer	30	11.7
	Merchant	95	37.1
	civil servant	33	12.9
Educational status	No formal education	92	35.9
	Primary	58	22.6
	Secondary	46	18.0
	Higher	60	23.5
Marital status	Married	211	82.4
	Single	23	8.9
	Widowed	14	5.5
	Divorced	8	3.2
FBS (mg/dl)	<110	27	10.5
	> = 110	229	89.5
Systolic blood pressure	<130	95	37.1
	> = 130	161	62.9
Diastolic blood pressure	<85	91	35.5
	> = 85	165	64.5
Dyslipidemic	Yes	115	44.9
	No	141	55.1
Body mass index(kg/m2	> = 30	66	23.5
	25–29.9	80	33.6
	18.5–24.9	71	27.7
	<18.5	39	15.2

### Behavioral characteristics of study participants

**Fig 1:**
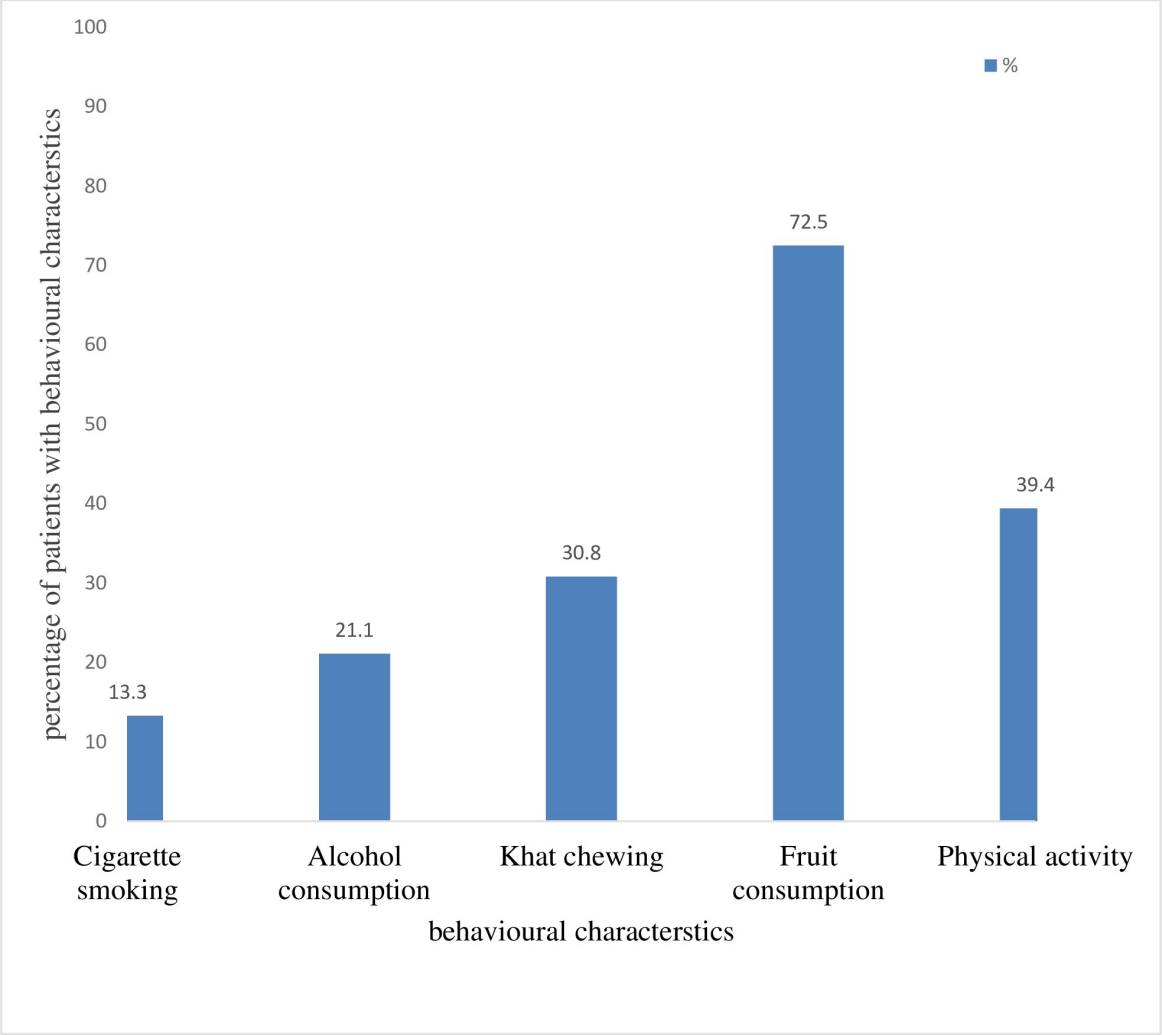
Behavioral factors among respondents admitted to medical ward with metabolic syndromes, in Jimma Medical Center, South west Ethiopia from April-May 2019.

All of patients took at least one form of medication during their hospital stay. Medications commonly used by patients were insulin and other anti-diabetics (63.3%), diuretics (42.6%), calcium channel blockers (34.0%) and ACE-inhibitors (31.6%). Nearly three quarter of patients were fruit consumers and those physically active were 39.4%. Among our study participants nearly one third was Khat chewer followed by alcohol consumer (21.1%) (Fig 1)

### Known comorbidities for electrolyte disturbance

**Fig 2:**
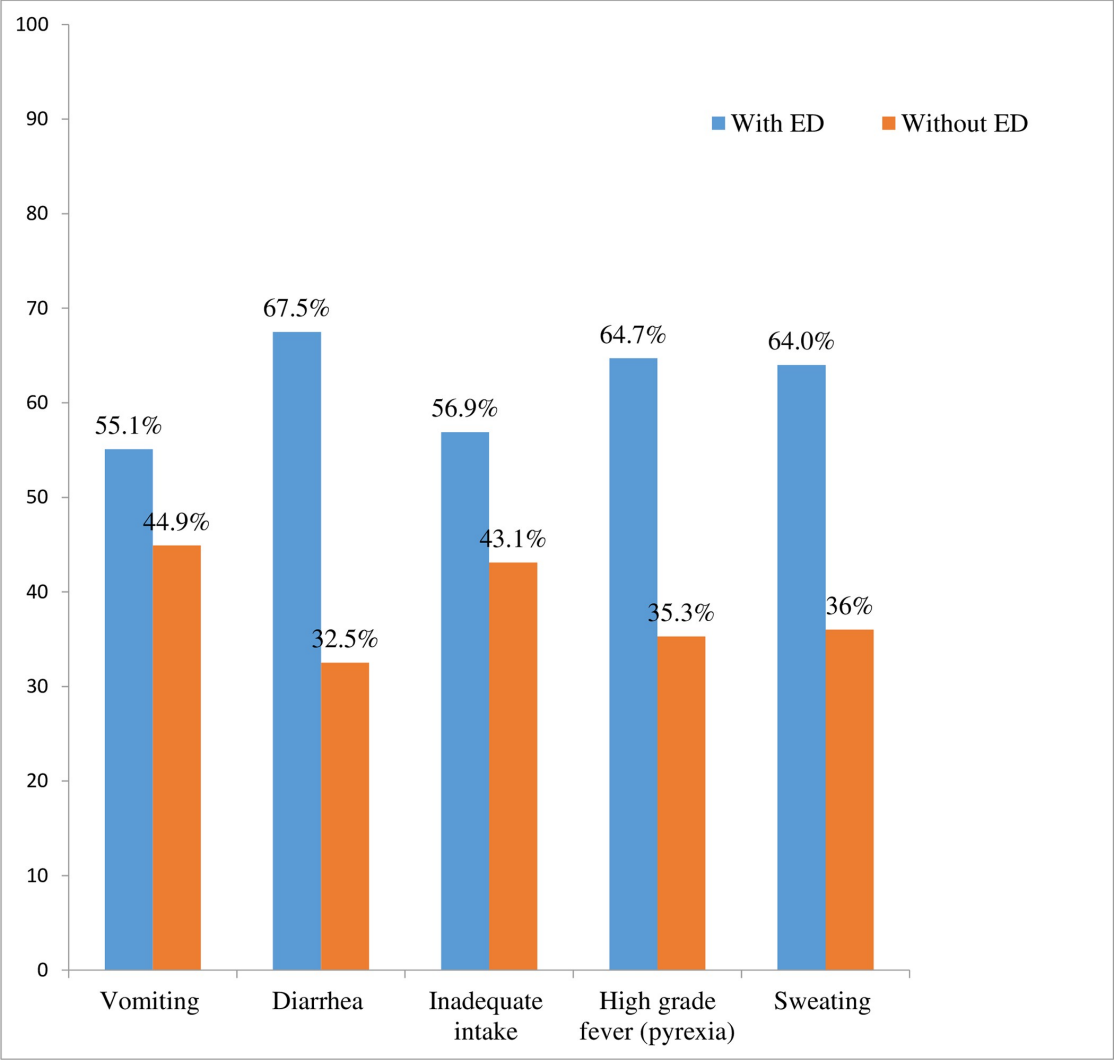
Distribution of known comorbidities precipitating for electrolyte disturbance among patients with and without electrolyte disturbance, in Jimma Medical Center, South west Ethiopia from April-May 2019.

Regarding to precipitating factors for electrolyte disturbance, majority (25.4%) of patients had sustained inadequate intake followed by vomiting (22.6%). Other precipitating factors for electrolyte disturbance complained were diarrhea (14.4%), sweating (9.7%) and high-grade fever (pyrexia) (6.6%) (Fig 2).

### Magnitude of electrolyte disturbance among study participants

Among a total of 256 study subjects, 113(44.1%) had at least one type electrolyte disturbance. Therefore; the magnitude of electrolyte disturbance was 44.1%(95%CI: 40.99–47.20). With regard to the pattern of electrolyte disorders as shown in Table 3, hyponatremia was found to be the commonest electrolyte disorder (42.9%) among the subjects under study. The mean serum sodium level of patients was 144.7mmol/L 95%CI (131.3–158.0 mmol/L). The second common electrolyte disturbance was hypokalemia (20.7%) and the mean serum level of potassium among study participants was 4.0 (SD±0.045mmol/L) (Table 2).

**Table 2:** Electrolyte status of patients admitted to medical ward with metabolic syndromes, in Jimma Medical Center, South west Ethiopia from April-May 2019.

Electrolytes	Category	Frequency	Percent
Sodium	Hyponatremia	110	42.9
	Normal	136	53.2
	Hypernatremia	10	3.9
Potassium	Hypokalemia	53	20.7
	Normal	194	75.8
	Hyperkalemia	9	3.5
Chlorine	Hypochloremia	45	17.6
	Normal	202	78.9
	Hypochloremia	9	3.5
Calcium	Hypocalcemia	24	9.5
	Normal	209	81.6
	Hypercalcemia	23	8.9

### Factors associated with electrolyte disturbance

Bivariate analysis was done and socio-demographic factors namely, patients age, residence, occupational status and educational status, were associated with electrolyte disturbance. In addition, factors namely alcohol consumption, diuretics, anti-diabetics and body mass index (BMI) were also found to be positively associated with electrolyte disturbances. Bivariate analysis was done for socio-demographic factors (age, residence, occupational status and educational status), which were associated with electrolytes disturbance. In addition, alcohol consumption, diuretics, anti-diabetics and body mass index were also found to be positively associated with electrolyte disturbances (Table 3).

**Table 3:** Bivariate analysis of factors associated with electrolyte disturbances among patients admitted to medical ward with metabolic syndromes, in Jimma Medical Center, South west Ethiopia from April-May 2019.

Variables	Categories		ED		p-value	COR (95% CI)
			Yes = 1	No = 0		
Age	> = 50		68	75	0.02	3.85(1.23,12.02) *
	25–49		41	51	0.39	3.41(1.07,10.94) *
	<25		4	17	1	
Sex	Female		62	61	0.53	1.63(0,92,2.69)
	Male		51	82	1	1
Residence	Urban		69	54	0.00	2.58(1.55,4.29) *
	Rural		44	89	1	1
Occupational status	Farmer		53	45	0.009	3.14(1.32,7.44) *
	Daily laborer		13	17	0.185	2.04(0.71,5.84)
	Merchant		38	57	0.194	1.78(0.74,4.24)
	civil servant		9	24		1
Educational status	No formal education		53	39	0.001	4.46(2.16,9.24) *
	Primary		28	30	0.005	3.07(1.39,6.75) *
	Secondary		18	28	0.082	2.11(1.43,4.90) *
	Higher		14	46	1	1
Marital status	Married		89	122	0.266	0.44(0.10,1.88)
	Single		15	8	0.890	1.12(0.21,5.97)
	Widowed		4	10	.129	0.24(0.04,0.51)
	Divorced		5	3	1	1
Cigarette smoking	Yes		16	18	0.713	1.14(0.55, 2.36)
	No		97	125	1	1
Habit of Alcohol consumption	Yes		37	17	0.00	3.61(1.90,6.85) *
	No		76	126	1	1
Vomiting	Yes		106	90	0.970	1.03(0.61, 1.66)
	No		32	28	1	1
Diarrhea	Yes		38	19	0.177	1.98(0.78,3.88)
	No		100	99	1	1
Fever	Yes		10	6	0.794	1.46(0.39,2.02)
	No		127	112	1	1
Sweating	Yes		10	6	0.29	1.46(0.08, 2.14)
	No		127	112	1	1
Inadequate intake	Yes		35	28	0.894	1.09(0.33,2.64)
	No		103	90	1	1
	Yes		38	41	0.394	1.26(0.74,2.15)
	No		75	102		
Fruit consumption	No		6	3	0.181	2.62(0.64,10.70)
	Yes		107	140	1	1
Physical activity	No		88	67	0.45	3.99(0,87,6.94)
	Yes		25	76	1	1
Medication	Diuretics	Yes	70	39	0.00	4.34(2.56,7.37) *
		No	43	104	1	1
	Insulin /anti DM	Yes	94	68	0.001	5.46(3.02,9.86) *
		No	19	75	1	1
	Ca-channel blockers	Yes	36	51	0.523	0.84(0.50,1.42)
		No	77	92	1	1
	ACE-inhibitors	Yes	34	47	0.635	0.88(0.51,1.50)
		No	79	96	1	1
Body mass index status (Kg/m^2^)	> = 30		35	25	0.001	6.40(2.44,16.81) *
	25–29.9		42	44	0.002	4.36(1.74,10.96) *
	18.5–24.9		29	42	0,017	3.15(1.23,8.12) *
	<18.5		7	32	1	1

*Variables significant at p-value less than 0.25.

*1 = Reference category*, *COR = Crud Odds Ratio*, *ED = electrolytes disorders*.

Multi variable logistic regression analysis was performed and educational status was found to be significantly associated with electrolyte disturbances. Patients with no formal education were nearly 7 times [AOR: 6.81;95%CI:(3.48,17.01)] more likely to sustained electrolyte disturbances than those patients with higher educational level. Behavioral characteristics namely, alcohol consumption also found to be significantly associated with electrolyte disturbances. Patients who took diuretics were more than 4 times [AOR: 4.39 95%CI (2.10, 9.15)] more likely to had electrolyte disturbances when compared to their counterparts (Table 4).

**Table 4:** Bivariate and multivariable logistic regression analysis of factors associated with electrolyte disturbances among patients admitted to medical ward with metabolic syndromes, in Jimma Medical Center, South west Ethiopia from April-May 2019.

Variables	Categories		ED		COR (95% CI)	AOR (95%CI)
			Yes = 1	No = 0		
Age	> = 50		68	75	3.85(1.23,12.02) *	3.55(0.50, 9.01)
	25–49		41	51	3.41(1.07,10.94) *	3.03(0.80, 8.17)
	<25		4	17	1	1
Residence	Urban		69	54	2.58(1.55,4.29) *	2.81(0.36, 5.82)
	Rural		44	89	1	1
Educational status	No formal education		53	39	4.46(2.16,9.24) *	6.81(3.48,13.01) **
	Primary		28	30	3.07(1.39,6.75) *	3.07(1.69,9.21) **
	Secondary		18	28	2.11(1.43,4.90) *	1.99(1.66,4.97) **
	Higher		14	46	1	1
Occupational status	Farmer		53	45	3.14(1.32,7.44) *	1.86(0.53,6.51)
	Daily laborer		13	17	2.04(0.71,5.84)	0.81(0.20,3.17)
	Merchant		38	57	1.78(0.74,4.24)	1.21(0.38,3.85)
	civil servant		9	24	1	1
Habit of Alcohol consumption	Yes		37	17	3.61(1.90,6.85) *	4.28(1.71,10.70) **
	No		76	126	1	1
Medication	Diuretics	Yes	70	39	4.34(2.56,7.37) *	4.39(2.10,9.15) **
		No	43	104	1	1
	Anti DM	Yes	94	68	5.46(3.02,9.86)	5.18(2.44,11.00) **
		No	19	75	1	1
Body mass index status (Kg/m2)	> = 30		41	25	7.49(2.88,19.52)	11.51(3.50,16.81) **
	25–29.9		36	44	3.74(1.47,9.47) *	9.52(2.61,12.67) **
	18.5–24.9		29	42	3.15(1.23,8.12) *	4.37(1.35,7.107) **
	<18.5		7	32	1	

* = Variable which had at p value<0.25 in bivariable analysis

** = Variables which had at p value <0.05 in multivariable analysis, *1 = Reference category*, *COR = Crud Odds Ratio*, *AOR = Adjusted Odds Ratio*, *ED = electrolytes disorder*.

## Discussion

Facility based cross-sectional study was conducted to assess the prevalence of electrolyte disorders among adults admitted to medical ward with Metabolic syndromes. The current study revealed that 44.1% of participants developed at least one electrolyte disorder with hyponatremia (42.9%), hypokalemia (20.7%), hypochloremia (17.6%) and hypocalcemia (9.4%). The finding was higher as compared to the reports from the community-based studies in Rotterdam where 15% of participants developed at least one form of electrolyte disturbance and in Nigeria where 5.5% of participants developed at least one electrolyte disturbance [2,3]. The disparity might be difference in socio demographic factors and the set up in which study participants were selected.

On the other hand, the finding was lower when compared to similar study done in Dhaka medical center where 78% of patients developed electrolyte disturbance [24]. This disparity might be explained by the socio-demographic variation between the study participants such as educational level and living standard as well as nature of the study area including better access to healthcare and information and health education. In addition, derangement of electrolyte balances may occur in admitted subjects with MetS, resulting from insulin deficiency, hyperglycemia, dyslipidemia, as a consequence of obesity, as well as administration of drugs such as diuretics, antidiabetic agents or exogenous insulin when compared to their counterparts [3,25,26].

In this study, the most common electrolyte abnormality seen was hyponatremia, which accounted in more than two fifth (42.9%) of the study participants. The finding was lower as compared to the reports from studies conducted in one of the largest hospitals of Bangladesh where 63.3% of participants were found to be hyponatremia and in Government Medical College of Dhaka where 80% of participants were found to be hyponatremia [24,27]. The disparity might be due to the fact that, the previous studies focused on subjects with renal problems or they focus on who are known to have a loss of renal-concentrating ability (electrolyte disturbance in admitted diabetes). On the other hand, the result was higher when compared to study done in a Multi-specialty Hospital in Southern Indians where 21.6% of patients were hyponatremic and Ethiopia where 28.9% of patients were hyponatremic [28,29]. The disparity might be due to the majority of participants in this were diabetics in which, patients have insulin resistance with uncontrolled blood sugar were at a greater risk to develop hyponatremia, since the inability of kidney to maintain control of homeostatic mechanisms involving stimulation of thirst, secretion of Antidiuretic Hormone (ADH), and renal handling.

Also subjects with urban dwellers have significantly associated hyponatremia. This might be related with physical inactivity due to lack of sufficient areas for physical exercise and related dietary changes. This in turn leads the dwellers to have higher BMI which is major cause of electrolyte derangement. In addition, comorbid conditions are commonly present with the geriatric population and associated with hyponatremia in elderly patients [30,31] and this supports our study that most of the patients had multiple co-morbid conditions.

Educational status also found to be associated with electrolyte disorder. Study subjects with no formal education were about 7 times [AOR: 6.81, 95%CI (3.48, 13.01)] more likely to developed electrolyte disorder when compared to those patients with higher educational status. This might be due to the fact that the educated ones are more likely to be aware of utilizing health services than uneducated ones. In addition, esucation is key to open any locked secrets of life in the world that enable every body to have standardized and healthy life style by getting a behavioral change.

The study also revealed that BMI had positively associated with electrolyte disorder [AOR: 4.37;95%CI (1.35,7.107)] and supported by similar studies conducted in India [17,32]. The possible explanation could be related with an increase in blood pressure, in which excessive adipose accumulation leads to increased circulating blood volume which results in higher cardiac output caused by stroke volume in obese patients and which could be due to hemodilution resulting from increased blood volume in high BMI or obesity [32]. Another study stated that an increase BMI (overweight and obese) can be one of the factors in increasing the blood pressure and at the same time may be responsible for lowering serum Na^+^ [33] which correlates with the findings of our study.

The study also identified that study subjects who had habit of alcohol consumption were more than four times [AOR: 4.28; 95%CI (1.71, 10.70)] more likely to had electrolyte disturbance than their counter parts. This finding was supported by similar studies [34,35]. The possible justifications could be, repeated exposure or prolonged consumption of alcohol results in increments of vasopressin levels, resulting in increased urine osmolality and decreased clearance of free water which might be resulted in hyponatremia by inducing water diuresis as short lived and followed by a period of fluid retention [36,37].

The other factor that showed significant association was taking diuretics in which the odds of developing electrolyte disorder were 4.39 higher among patients with diuretics when compared to their counterparts. This was in line with studies conducted in India, Switzerland and Ethiopia [38–40]. This might be dues to the fact that drugs especially diuretics are a major cause of hyponatremia in DM and hypertensive patients.

Patients with anti-diabetic treatments were 5.18 times more likely to had electrolyte disorder than their counter parts. The possible explanation could be, well known that the severe hyperglycemia in DM can cause osmotic diuresis leading to dehydration and electrolyte loss, particularly sodium, chloride and magnesium. Dehydration in turn induces secondary hyper-aldosteronism that exacerbates K^+^ loss. As a result, patient with DM may have hypokalemia. Potassium facilitates the function of insulin in the delivery of glucose to cells; when insulin binds to its receptors on the cell membrane, it causes K+ flow into the cells. In diabetics, excessive use of insulin is associated with hypokalemia; those with low blood glucose and hypokalemia should avoid using insulin. Routine outpatient insulin treatment does not cause significant hypokalemia [41,42].

Despite the fact that the necessary endeavors were made to minimize or avoid the possible limitations of this study, the result should be interpreted in the light of the following unavoidable limitations. There might be a possibility of recall bias since participants were asked for events which have already happened within the past prior to this study. Causality cannot be inferred due to the cross-sectional nature of the study. The study might be among few studies which tried to assess the level of electrolyte disorder in general. In addition, since the study was conducted among admitted patients, which can not be generalized to the out patient attendants.

## Conclusion

The finding from the study revealed that nearly half the study participants with MetS had electrolyte disorder. Hyponatremia and hypokalemia were the predominant electrolyte disorders. Variations in electrolyte status were noticed between patients with co-morbidity and their counterparts. Variables namely, no formal education, alcohol consumption, diuretics, anti-diabetics and BMI were found to be significantly associated with electrolyte disorder in multi variable logistic regression. Supporting information.

## Supporting information

S1 Data (SAV)
